# ANCA-negative eosinophilic granulomatosis with polyangiitis complicated by peripheral nerve damage: A case report

**DOI:** 10.1097/MD.0000000000034450

**Published:** 2023-08-04

**Authors:** Yongzhen Chen, Qiuxia Wan, Bo Liu

**Affiliations:** a Department of Neurology, Shenzhen Longhua District Central Hospital, Shenzhen, China; b Department of Hematology, Shenzhen Longhua District Central Hospital, Shenzhen, China.

**Keywords:** ANCA-associated vasculitis, Churg-Strauss syndrome, eosinophilic granulomatosis with polyangiitis, gastroenteritis, peripheral neuropathy

## Abstract

Eosinophilic granulomatosis with polyangiitis (EGPA) is a rare autoimmune disease that can affect multiple systems of the body and is characterized by asthma, blood and tissue eosinophilia, and small vascular inflammation. Eosinophilic tissue infiltration and extravascular granuloma formation can lead to any organ damage, but peripheral neuropathy is relatively rare.

**Patient concerns:**

A 29-year-old male patient was admitted to the hospital due to fever and rash on both lower extremities for 18 days. The patient complained of muscle pain in both lower extremities, with nausea, anorexia, abdominal pain, and diarrhea. He had a 2-year history of asthma and bronchiectasis. The physical examination results were as follows: temperature, 37.8 °C; multiple patchy red rashes on both lower extremities; and no obvious abnormalities in other systems. The patient was negative for anti-neutrophil cytoplasmic antibody (ANCA). Chest computed tomography showed bilateral ground-glass opacities, small nodules, and bronchiectasis. Histopathology of rectal tissues revealed numerous eosinophilic infiltrations. One week after admission, the patient developed symptoms of peripheral nerve damage, presenting with distal weakness in both lower extremities, foot drop, cross-threshold gait, and hypoalgesia on the lateral sides of both lower legs. Electromyography showed that the motor sensory fibers of the lower extremities were damaged.

**Diagnoses:**

Referring to the diagnostic criteria of the American College of Rheumatology in 1990, the patient was diagnosed with systemic EGPA (vasculitic phase) with rare peripheral nerve damage.

**Interventions:**

After diagnosis, the patient was administered oral prednisone (60 mg/d; 1.0 mg/kg/d), and cyclophosphamide (900 mg) was infused on the 5th and 18th days of hormone therapy. Prednisone was reduced to 50 mg/d 1 month thereafter.

**Outcomes:**

After 1+ months of treatment, most of the symptoms disappeared. Limb weakness did not improve. Currently, the patient is undergoing outpatient follow-up and is adhering to treatment.

**Lessons:**

EGPA is a rare disease that can affect multiple systems and has diverse clinical manifestations, with no specific manifestations in the early stage. Diagnosis is difficult, and there is a high misdiagnosis rate. The rate of ANCA positivity for this disease is not high, and clinicians should consider the possibility of ANCA-negative EGPA.

## 1. Introduction

Eosinophilic granulomatosis with polyangiitis (EGPA) is a rare autoimmune disease that can affect multiple systems in the body. It is characterized by asthma, blood and tissue eosinophilia, and small vascular inflammation. Eosinophilic tissue infiltration and extravascular granuloma formation can lead to damage in any organ and can cause lung infiltrates, sinus disease, peripheral neuropathy, renal and cardiac involvement, and skin rashes. In 1951, Churg and Strauss first proposed allergic granulomatous vasculitis, that is, Churg-Strauss syndrome. The incidence of this disease is extremely low, the clinical symptoms are complex, onset is insidious, and the disease course is long, making it difficult to diagnose. Asthma is often the first symptom of EGPA, but EGPA can often take a long time to diagnose. This article reports a case of EGPA with fever, rash, peripheral nerve damage, and proctitis as the main manifestations. The patient had several visits to the departments of respiratory medicine, dermatology and hematology. We are reporting this case to provide a reference for the clinical treatment and diagnosis of EGPA.

## 2. Case report

**Figure 1.**
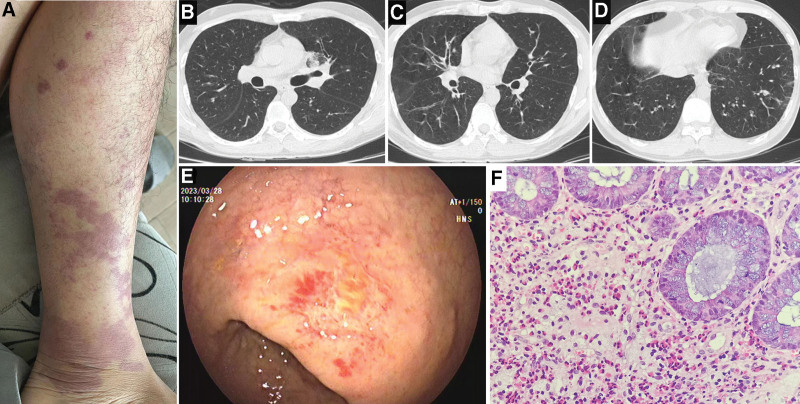
(A) The patient presented multiple patchy rashes on both lower limbs; the rashes were dark red in color, with no tenderness, no itching, no nodules, and no blisters. (B–D) Chest CT showed bilateral ground-glass opacities, small nodules, and bronchiectasis. (E) The gastrointestinal endoscopy diagnosis was chronic superficial gastritis and multiple rectal ulcers. (F) The pathological diagnosis was focal (rectal) infiltration of numerous eosinophils (HE, ×400). CT = computed tomography.

The patient, male, 29 years old, had a fever without an obvious cause on March 3, 2023; his highest temperature was 38.5°C. Additionally, the patient presented multiple patchy rashes on both lower limbs; the rashes were dark red in color, with no tenderness, no itching, no nodules, and no blisters (Fig. 1A). This was accompanied by muscle soreness in both lower extremities, which was aggravated during walking and weight bearing; nausea, anorexia, abdominal pain, and diarrhea were noted. On March 6, 2023, he went to the outpatient dermatology department of our hospital for treatment. The results of a routine blood test were as follows: white blood cells, 15.68 × 10^9^/L; red blood cells, 7.54 × 10^12^/L; neutrophils, 9.46 × 10^9^/L; eosinophils, 3.79 × 10^9^/L; percentage of eosinophils, 24.2%; and high-sensitivity C-reactive protein, 9.79 mg/L. After treatment with azelastine hydrochloride tablets, clindamycin palmitate hydrochloride dispersible tablets, and hydrocortisone butyrate cream, the fever resolved, and the rash subsided. On March 20, 2023, the above symptoms, such as fever, rash, and muscle pain, recurred. The patient was admitted to the hematology ward of our hospital for treatment on March 21, 2023 with the chief complaint of “fever with rash on both lower extremities for 18 days.” The patient had a history of asthma and bronchiectasis for 2 years and visited the outpatient respiratory medicine department of our hospital many times. The results of the physical examination on admission were as follows: temperature, 37.8°C; pulse, 123 beats/min; respiration, 22 breaths/min; BP, 102/66 mm Hg; multiple patchy red rashes on both lower extremities; locally increased skin temperature; no tenderness; and no skin ulceration. Other system examinations showed no obvious abnormalities. The diagnoses on admission were eosinophilia and skin and soft tissue infection. The results of a repeat routine blood test on March 22, 2022 were as follows: white blood cells, 23.93 × 10^9^/L; red blood cells, 7.39 × 10^12^/L; platelets, 327 × 10^9^/L; eosinophils, 11.42 × 10^9^/L; percentage of eosinophils, 47.7%; high-sensitivity C-reactive protein, 49.93↑mg/L; erythrocyte sedimentation rate, 20.00↑mm/h; interleukin 6, 46.7↑pg/ml; alanine aminotransferase, 97↑U/L; aspartate aminotransferase, 36 U/L; γ-glutamyltransferase, 186↑U/L; and alkaline phosphatase, 152↑U/L. Electrocardiogram revealed sinus tachycardia. Cardiac color ultrasound revealed no abnormalities in cardiac morphology and structure; left ventricular systolic and diastolic functions were normal. The chest computed tomography (CT) findings included bronchiectasis with peripheral inflammation in both lungs; subpleural infectious lesions in the lateral and inner anterior basal segments of the left lower lobe; and ground-glass opacities and multiple small nodules in both lungs (Fig. 1B–D). Abdominal CT revealed that the rectum was slightly thickened locally. The results of bone marrow smear cytology on March 24, 2023, were as follows: eosinophilic myelocytes, 13.0↑%; eosinophilic metamyelocytes, 8.5↑%; and eosinophilic rod granulocytes, 6.0↑%. Myeloid leukemia common fusion gene screening was negative. The results of a flow cytometry immunofluorescence analysis were as follows: no obvious evidence of abnormal immunophenotypes related to acute leukemia, non-Hodgkin’s lymphoma and high-risk myelodysplastic syndrome and approximately 32.1% eosinophils. The bone marrow biopsy results were as follows: active proliferation of bone marrow nucleated cells (approximately 60% hematopoietic volume), slightly higher granulocyte to erythrocyte ratio, no typical ALIP and hot spots; and myeloid hyperplasia, mainly cells in the mature stage, with clear visualization of eosinophils. During hospitalization, the patient was administered oral prednisone (0.5 mg/kg/d) and allopurinol. The patient’s fever resolved, rash subsided, and muscle pain decreased; overall, the patient’s condition improved.

One week after admission, the patient developed symptoms of distal weakness in both lower extremities, foot drop, cross-threshold gait, hypoalgesia on the lateral sides of both calves, headache, nausea, anorexia, abdominal pain, diarrhea and other gastrointestinal discomfort symptoms. On March 28, 2023, blood test revealed the following: total IgE > 2500.00 IU/mL; rheumatoid factor, 896.4 IU/mL; proteinase 3-anti-neutrophil cytoplasmic antibody (ANCA), negative; MPO-ANCA, negative; p-ANCA, negative; and c-ANCA, negative. Sinus CT indicated inflammation of the bilateral maxillary sinuses and ethmoid sinuses and nasal septum deviated to the right and hypertrophied left inferior turbinate. Brain magnetic resonance imaging revealed no obvious abnormalities. The following electromyography results were obtained: for motor nerve conduction velocity, the compound muscle action potential amplitude of the left common peroneal nerve was not detected (record of the extensor digitorum brevis), the compound muscle action potential amplitude of the right common peroneal nerve was low, and the motor nerve conduction velocity of the left and right common peroneal nerves was normal; and for the sensory nerve conduction velocity, the sensory nerve action potential amplitude of the left and right common peroneal nerves was low, and the sensory nerve conduction velocity of the left and right common peroneal nerves was slightly slow. These findings led to the conclusion that the motor and sensory fibers of both lower extremities were damaged. On, April 1, 2023, a lumbar puncture cerebrospinal fluid examination was performed, with the following results: pressure, 120 mm H_2_O; cerebrospinal fluid, colorless and transparent; no clot; Pandy’s test, negative; total number of nucleated cells, 8 × 10^6^; percentage of mononuclear cells, 19.60%; percentage of multi-nucleated cells, 80.40%, no red blood cells and no pus cells were found in a microscopic examination; glucose, 2.86 mmol/L; chlorine, 118 mmol/L; and micro total protein, <100.00 mg/L. The gastrointestinal endoscopy diagnosis was chronic superficial gastritis and multiple rectal ulcers (Fig. 1E). The pathological diagnosis was focal (rectal) infiltration of numerous eosinophils (Fig. 1F).

The patient presented with fever, rash, and muscle pain in both lower extremities. In addition to the significant increase in the absolute value and percentage of eosinophils in the patient’s peripheral blood, lung CT showed ground-glass opacities, small nodules, and bronchiectasis in both lungs. Colonoscopy revealed multiple rectal ulcers, pathology revealed focal eosinophilic infiltration, and sinus CT showed bilateral maxillary sinus and ethmoid sinus inflammation. The patient had a history of asthma and clear peripheral nerve damage. Referring to the diagnostic criteria of the American College of Rheumatology in 1990,^[1]^ the patient was diagnosed with systemic EGPA (vasculitic phase) with rare peripheral nerve damage. After diagnosis, the patient was administered oral prednisone (60 mg/d, 1.0 mg/kg/d), and cyclophosphamide (900 mg) was infused on the 5th and 18th days of hormone therapy. After 1 month, the prednisone was reduced to 50 mg/d. The patient had no recurrence of fever, the rash subsided, and there were no symptoms of gastrointestinal discomfort after 1 + month of treatment. Laboratory tests indicated that the eosinophil count was close to the normal range. Additionally, the degree and frequency of pain in the limbs significantly decreased, and the numbness resolved; however, limb weakness did not improve. Currently, the patient is undergoing outpatient follow-up and is adhering to treatment.

## 3. Discussion

EGPA was first named Churg-Strauss syndrome by pathologists Churg and Straus in 1951 and was renamed EGPA in 2012. It is the rarest type of anti-neutrophil cytoplasmic antibody-associated vasculitis. According to studies, the prevalence rate of EGPA is 10.7 to 17.8/million people, and the incidence rate is 0.9 to 2.4 new cases/million people per year. There are no significant sex differences, family aggregation, or racial tendencies. It is a rare and difficult-to-diagnose disease, with hidden and complex clinical manifestations, leading to misdiagnosis and missed diagnosis.^[2]^

The etiology of EGPA is unknown but may be related to environmental, genetic and immune factors. At present, the pathogenesis is believed to be mainly ANCA-mediated vascular wall damage and eosinophil infiltration-mediated damage.^[3]^ Studies have shown that ANCA can activate neutrophils to generate reactive oxygen species and release lysosomal proteolytic enzymes, which can affect the function of vascular endothelial cells and increase vascular permeability.^[4]^ Clinically, 30% to 50% of patients with EGPA are ANCA positive, with MPO-ANCA accounting for 71.4% to 100% of ANCA-positive cases. Based on whether a patient is ANCA positive or not, EGPA is divided into 2 types: vasculitic (ANCA positive) and nonvasculitic (ANCA negative).^[5]^ Vasculitic EGPA has kidney involvement but is also associated with purpura, alveolar hemorrhage, sinusitis, etc., and the incidence of peripheral neuropathy is high. In contrast, nonvasculitic EGPA has pulmonary involvement, with a greater incidence of cardiac involvement (e.g., pericarditis and cardiomyopathy), pleural effusion, gastrointestinal involvement and fever.^[6]^ A recent genome-wide association study showed that EGPA can be classified into 2 subgroups that are genetically and clinically distinct, with the ANCA-positive subgroup possessing major histocompatibility complexes similar to those of microscopic polyangiitis (MPA) patients and the ANCA-negative subgroup having genetics (GPASS and IL-5) similar to those of asthma and inflammatory bowel disease patients.^[7]^ However, the impact of ANCA-negative and ANCA-positive status on the treatment strategy for and prognosis of patients with EGPA is still controversial, and further studies with larger sample sizes and long-term follow-up are needed.

The natural course of EGPA can be divided into the prodromal phase, eosinophilic infiltration phase and vasculitic phase. The prodromal phase is mainly characterized by asthma and allergic rhinitis, often accompanied by sinusitis and nasal polyps; as the disease progresses, eosinophils infiltrate the tissues of organs, for example, the lungs, heart and gastrointestinal tract, manifesting as nonfixed lung shadows, myocardial involvement, abdominal pain or gastrointestinal bleeding and even perforation. The most common manifestation of the vasculitic phase is mononeuritis multiplex, with renal and cutaneous involvement also occurs in this stage. Notably, there are no clear demarcations for disease staging, and patients may present with wheezing, eosinophilic infiltration, and vasculitis simultaneously. Studies have shown that the existence of purpura and the Birmingham vasculitis activity score for EGPA are significantly correlated, indicating that skin lesions indicate high EGPA activity.^[5]^

Pathological examination is considered to be the gold standard for diagnosing EGPA, and samples derived from skin, muscle, nerve, gastrointestinal tract, lung and other tissues can be used for such examinations. EGPA has 3 typical pathological features: necrotizing vasculitis with eosinophilic infiltration of surrounding tissue and extravascular granulomas. The pathological changes in EGPA are usually staged, and the pathological manifestations are dependent on the biopsy site and the disease phase. Therefore, it is rare for the 3 typical pathological features to coexist in the same biopsy tissue specimen. The current histopathological diagnostic criteria stipulate that there must be evidence of extravascular eosinophils. However, studies have shown that eosinophilic infiltration is only found in 58% of skin lesion specimens and that approximately 67% of the samples have evidence of vasculitis and 16% of the specimens have evidence of extravascular granulomas.^[8]^ Some scholars have suggested that vasculitis and extravascular granuloma formation can also be used as histopathological diagnostic criteria when the clinical criteria for EGPA are met in order to avoid delays in diagnosis.^[9]^ At present, the diagnosis of EGPA is mainly based on the criteria of the American College of Rheumatology in 1990^[1]^: asthma-like symptoms; peripheral blood eosinophil differential count > 10%; single or multiple neuropathy; paranasal sinus disease; X-ray showing migratory infiltration shadows in the lungs; and tissue biopsy confirming extravascular eosinophilic infiltration. Four out of 6 are needed to confirm the diagnosis of EGPA, with a sensitivity of 85.0% and a specificity of 99.7%. EGPA can be divided into 2 types: localized and systemic. Patients with EGPA who meet at least 4 of the 6 criteria established by the American College of Rheumatology in 1990 and only have lung and respiratory system involvement (including ear-nose-throat) are considered to have localized EGPA. Patients having at least 2 or more organs involved are considered to have systemic EGPA. Localized EGPA can transition into systemic EGPA. The patient in this report had a history of asthma, the differential count of eosinophils in peripheral blood was 47.7%, electromyography showed peripheral neuropathy, sinus CT showed sinusitis, and chest CT showed bilateral lung bronchiectasis with peripheral inflammation and ground-glass opacities in the lungs. Rectal histopathology revealed a large amount of eosinophilic infiltration, and other rheumatic immune and hematological diseases were excluded, thus meeting the above diagnostic criteria for systemic EGPA (vasculitic phase).

EGPA needs to be differentiated from the following diseases. Granulomatosis with polyangiitis, formerly known as Wegener’s granulomatosis, is a necrotizing granulomatous vasculitis, and the upper and lower respiratory tract and kidneys are usually involved. Granulomatosis with polyangiitis is not associated with wheezing-like symptoms, there is no apparent increase in peripheral blood eosinophils, and c-ANCA and/or proteinase 3-ANCA are present. MPA is systemic necrotizing vasculitis mainly involving small blood vessels, which can invade organs such as the kidneys, skin, and lungs. MPA is not associated with obvious wheezing symptoms, there is no significant increase in peripheral blood eosinophils, p-ANCA and/or MPO-ANCA is present, and there are no eosinophilic infiltration and granulomatous lesions in histopathology. Polyarteritis nodosa is necrotizing vasculitis involving medium and small arteries, mostly with rash and peripheral nervous system damage, without typical clinical manifestations of asthma. The proportion of eosinophils in peripheral blood is not significantly increased, ANCA is not present, and pathological biopsy indicates nongranulomatous vasculitis. Hypereosinophilic syndrome (HES) is characterized by increased eosinophilia and multisystem involvement, but eosinophil counts in HES are often higher than those in EGPA. The reason for multisystem involvement is eosinophil infiltration; therefore, there are almost no pathological changes in vasculitis and granulomas, and delayed-onset asthma rarely occurs. Nervous system involvement is more common in the peripheral nerves in EGPA patients and more common in the central nervous system in HES patients. Vascular involvement is mainly vasculitis in EGPA patients and mostly arteriovenous thrombosis in HES patients.

Treatment for patients with EGPA depends on factors such as disease severity, involved organs, and disease activity. At present, disease severity and patient prognosis of patients are mainly determined using the 5-factor score (FFS) developed by the French Vasculitis Study Group^[10]^: central nervous system involvement; digestive tract involvement; myocardium involvement; 24 hours proteinuria > 1 g; and renal insufficiency (serum creatinine > 140 μmol/L). Each of the above 5 criteria is worth 1 point; when the FFS is 0 points, glucocorticoids can be used alone, and when the FFS is ≥1 point or patients have severe organ involvement (including severe peripheral neuropathy, severe ocular disease, alveolar hemorrhage and/or glomerulonephritis) treatment should include hormones combined with immunosuppressants (such as cyclophosphamide) to induce remission. There is still no concensus on the duration of remission induction therapy. The 2015 global expert consensus on the management of EGPA recommends a treatment duration of at least 24 months after remission has been achieved.^[11]^ Other treatments, including plasma exchange, intravenous immunoglobulins, and interferons, are all effective. In recent years, the application prospects of targeted therapy drugs, such as mepolizumab and rituximab, have been good.^[12]^ According to the FFS scoring standard, the patient had clear evidence of gastrointestinal involvement, that is, FFS = 1; therefore, it was reasonable to choose glucocorticoids + immunosuppressants (cyclophosphamide) for treatment, and the patient’s clinical symptoms significantly improved. EGPA has a relatively poor prognosis, with a recurrence rate of 30% to 40% and a mortality rate of 5% to 10%. The prognosis depends on whether an early diagnosis is obtained and timely treatment is administered.^[5]^ The leading causes of death are heart failure and myocardial infarction, followed by renal failure, gastrointestinal perforation and intracranial hemorrhage. Those with frequent asthma attacks and rapid progression of systemic vasculitis have a poor prognosis.^[13]^

The clinical manifestations of EGPA patients are complex, and early identification is difficult; it can involve multiple systems and organs, such as the lungs, sinuses, skin, nervous system, digestive system, heart, and kidneys. Because EPGA involves the nervous system, clinicians in neurology departments should have a good understanding of this disease. When peripheral neuropathy occurs with asthma or allergic rhinitis, EGPA should be considered. Clinicians should conduct routine blood tests, bone marrow puncture, cranial magnetic resonance imaging, neurophysiological examinations and other related examinations to facilitate early diagnosis and administer early treatment to improve the prognosis of patients.

## 4. Conclusion

EGPA is a rare disease that can affect multiple systems and has diverse clinical manifestations, with no specific manifestations in the early stage. Diagnosis is difficult, and there is a high misdiagnosis rate. In this case, if a dermatopathological examination had been administered and revealed evidence of eosinophilic infiltration, an earlier diagnosis may have been obtained. The rate of ANCA positivity for this disease is not high, and clinicians should consider the possibility of ANCA-negative EGPA. Individualizing treatment is recommended to improve prognosis.

## Author contributions

**Conceptualization:** Yongzhen Chen, Qiuxia Wan.

**Data curation:** Yongzhen Chen, Qiuxia Wan.

**Formal analysis:** Yongzhen Chen, Qiuxia Wan.

**Investigation:** Yongzhen Chen, Qiuxia Wan.

**Methodology:** Bo Liu.

**Supervision:** Bo Liu.

**Writing – original draft:** Yongzhen Chen.

**Writing – review & editing:** Bo Liu.

## References

[1] Masi A. The American College of Rheumatiology 1990 criteria for the classification of Churg-Strauss syndrome (allergic grnulomatosis and angitis). Arthritis Rheum. 1990;33:1094–100. 2202307 10.1002/art.1780330806

[2] Furuta S Iwamoto T Nakajima H. Update on eosinophilic granulomatosis with polyangiitis. Allergol Int. 2019;68:430–6. 31266709 10.1016/j.alit.2019.06.004

[3] Imre N Strek ME Leff AR. Churg-Strauss syndrome. Lancet. 2003;361:587–94. 12598156 10.1016/S0140-6736(03)12518-4

[4] Hong X Peter H Peiqi H. Antineutrophil cytoplasmic autoantibodies specific for myeloperoxidase cause glomerulonephritis and vasculitis in mice. J Clin Investig. 2002;110:955. 12370273 10.1172/JCI15918 PMC151154

[5] Kataoka H Tomita T Kondo M. Presence of purpura is related to active inflammation in association with IL-5 in eosinophilic granulomatosis with polyangiitis. Rheumatol Int. 2021;41:449–54. 32770271 10.1007/s00296-020-04672-8 PMC7835155

[6] Abril A. Churg-Strauss syndrome: an update. Curr Rheumatol Rep. 2011;13:489–95. 21863326 10.1007/s11926-011-0205-7

[7] Lyons PA Peters JE Alberici F. Genome-wide association study of eosinophilic granulomatosis with polyangiitis reveals genomic loci stratified by ANCA status. Nat Commun. 2019;10:5120. 31719529 10.1038/s41467-019-12515-9 PMC6851141

[8] Bosco L Peroni A Schena D. Cutaneous manifestations of Churg-Strauss syndrome: report of two cases and review of the literature. Clin Rheumatol. 2011;30:573–80. 20949297 10.1007/s10067-010-1593-1

[9] Bridges C Shenk M Martin K. Cutaneous manifestations of childhood eosinophilic granulomatosis with polyangiitis (cEGPA): a case-based review. Pediatr Dermatol. 2020;37:604–12. 32212191 10.1111/pde.14144

[10] Guillevin L Pagnoux C Seror R.; French Vasculitis Study Group (FVSG). The five-factor score revisited: assessment of prognoses of systemic necrotizing vasculitides based on the French Vasculitis Study Group (FVSG) cohort. Medicine (Baltimore). 2011;90:19–27. 21200183 10.1097/MD.0b013e318205a4c6

[11] Groh M Pagnoux C Baldini C. Eosinophilic granulomatosis with polyangiitis (Churg-Strauss) (EGPA) Consensus Task Force recommendations for evaluation and management. Eur J Intern Med. 2015;26:545–53. 25971154 10.1016/j.ejim.2015.04.022

[12] Ford JA Aleatany Y Gewurz-Singer O. Therapeutic advances in eosinophilic granulomatosis with polyangiitis. Curr Opin Rheumatol. 2022;34:158–64. 35440531 10.1097/BOR.0000000000000873

[13] White J Dubey S. Eosinophilic granulomatosis with polyangiitis: a review. Autoimmun Rev. 2023;22:103219. 36283646 10.1016/j.autrev.2022.103219

